# Cost-Effectiveness of Bariatric Surgery in Patients Living with Obesity and Type 2 Diabetes

**DOI:** 10.1155/2023/9686729

**Published:** 2023-12-16

**Authors:** Gábor Kovács, Elemér Mohos, János Tibor Kis, Ádám Tabák, Péter Gerendy, Judit Pettkó, Dávid Nagy, Dávid Győrbíró, Zoltán Kaló

**Affiliations:** ^1^Syreon Research Institute, Budapest, Hungary; ^2^Department of General Surgery Territory Hospital Veszprém, Hungary; ^3^Department of Internal Medicine Centrum, Szent János Hospital, Budapest, Hungary; ^4^Department of Internal Medicine and Oncology, Semmelweis University Faculty of Medicine, Budapest, Hungary; ^5^Department of Public Health, Semmelweis University Faculty of Medicine, Budapest, Hungary; ^6^UCL Brain Sciences, University College London, London, UK; ^7^National Health Insurance Fund Management, Budapest, Hungary; ^8^European Coalition for People Living with Obesity, Dublin, Ireland; ^9^Center for Health Technology Assessment, Semmelweis University, Budapest, Hungary

## Abstract

**Aims:**

The favourable effects of bariatric surgeries on body weight reduction and glucose control have been demonstrated in several studies. Additionally, the cost-effectiveness of bariatric surgeries has been confirmed in several analyses. The aim of the current analysis was to demonstrate the cost-effectiveness of bariatric surgeries in obese patients with type 2 diabetes in Hungary compared to conventional diabetes treatments based on economic modelling of published clinical trial results.

**Materials and Methods:**

Patients entered the simulation model at the age of 45 with *body* *mass* *index* (*BMI*) ≥ 30 kg/m^2^ and type 2 diabetes. The model was performed from the public payer's perspective, comparing sleeve gastrectomy (SG) and Roux-en-Y gastric bypass (RYGB) procedures to conventional care of diabetes. The results were provided separately for three BMI categories.

**Results:**

The base-case analysis demonstrated that both surgery types were dominant; i.e., they saved 17 064 to 24 384 Euro public payer expenditures and resulted in improved health outcomes (1.36 to 1.50 quality-adjusted life years gain (QALY)) in the three BMI categories. Bariatric surgeries extended the life expectancy and the disease-free survival times of all the investigated diabetes complications. All the scenario analyses confirmed the robustness of the base-case analysis, such that bariatric surgeries remained dominant compared to conventional diabetes treatments.

**Conclusion:**

The results of this cost-effectiveness analysis highlight the importance of bariatric surgeries as alternatives to conventional diabetes treatments in the obese population. Therefore, it is strongly recommended that a wider population has access to these surgeries in Hungary.

## 1. Introduction

In a recently published document, the WHO defines overweight and obesity as a body mass index (BMI) ranging from ≥25 kg/m^2^ to <30 kg/m^2^ and from ≥30 kg/m^2^, respectively [1]. The Centers for Disease Control and Prevention has subdivided obesity into classes 1 to 3 according to BMI levels (as ≥30 to <35, ≥35 to <40, and ≥40 kg/m^2^, respectively) [2]. The age-standardized global prevalence of obesity has increased in the last 40 years from 4.6% to 14.0% [3]. Moreover, the highest prevalence was observed in the Americas and Europe (with rates of 22.4% and 20.0% in 2019, respectively). The West-Pacific and South-East Asian regions showed the lowest prevalence of obesity over the entire 40-year observation period, although an increase could be detected in all regions throughout the world [3].

Based on Eurostat data, the lowest prevalence of obesity in Europe was observed in Romania in both 2014 and 2019, whereas the highest prevalence was observed in Malta in both years (25.2% and 28.7%, respectively), followed by Hungary (20.6% and 24.5%, respectively) [4]. Obesity is most frequently observed in the age range of 50-59 years (with a peak of 27% observed in females and 20% observed in males), whereas it is lower in both younger and older populations [3].

In Hungary, the prevalence of the combination of diabetes and obesity (*BMI* ≥ 30 kg/m^2^) is 3.5% in the population aged ≥15 years (1.5% for patients with diabetes and *BMI* ≥ 35 kg/m^2^ and 0.43% for those with *BMI* ≥ 40 kg/m^2^, irrespective of the type of diabetes). This indicates that approximately 170,000 class 1, 88,000 class 2, and 36,000 class 3 obese patients can be expected in the ≥15-year age population in Hungary. If only the 35- to 64-year-old obese and diabetic patients are considered, the estimated numbers of the obese patients with T2DM are approximately 89 000, 51 100, and 25 500, respectively (based on European Health Interview Survey in 2014 and demographic data) [5, 6]. Obesity can lead to a wide range of complications, including uncontrolled glucose metabolism, fatty liver and gallbladder diseases, joint diseases, and an increased risk of some types of cancers [7].

The recently published clinical practice guidelines of the European Association for Endoscopic Surgery (EAES) recommend bariatric surgery for all patients with a *BMI* ≥ 40 kg/m^2^, for patients with associated comorbidities in the ≥35 to 40 kg/m^2^ BMI range, and for patients with refractory type 2 diabetes or hypertension in the ≥30 to 35 kg/m^2^ range [8]. The guidelines prefer sleeve gastrectomy (SG) and Roux-en-Y gastric bypass (RYGB) to banding. In the RYGB versus SG comparison, both procedures offer similar weight loss and DM control, but RYGB may be preferred to SG in severe gastroesophageal reflux disease (GERD). The Society of American Gastrointestinal and Endoscopic Surgeons (SAGES) states a similar recommendation on the indication of metabolic surgeries [9].

The two main types of bariatric surgeries are restrictive and primarily malabsorptive procedures, with the former surgery limiting the gastric volume but not rerouting the food pathway and the latter surgery excluding some part of the small intestine and reducing the area of the mucosa available for absorption [10]. Restrictive procedures include adjustable banding (AGB) and sleeve gastrectomy (SG), and malabsorptive procedures include Roux-en-Y gastric bypass (RYGB) and biliopancreatic diversion (BPD) with or without duodenal switch (DS). In addition to offering weight loss, bariatric surgeries also improve glycaemic control through the following three mechanisms: weight loss/decreased food intake, intestinal malabsorption, and changes in the dynamics of intestinal hormones due to the bypass of a part of the gut [11]. Although the first two mechanisms can decrease hyperglycaemia, experiments have suggested that the bypass of the proximal small intestine (in malabsorptive procedures) has the most effect on glycaemic control [11]. Bypass procedures exert long-term effects (among others) via enhanced beta-cell function and increased peripheral insulin sensitivity [12].

The outcomes of bariatric surgeries can be classified as short-, medium-, and long-term effects [13]. From a cost-effectiveness perspective, medium- and long-term consequences have the most considerable importance. Several reviews have investigated the aggregated medium- and long-term effects of bariatric surgeries. Yu et al. [14] demonstrated (based on the pooled data of 26 studies) that bariatric surgeries were associated with a 50% loss of excess weight, a 13.4 kg/m^2^ reduction in BMI, and a 1.8% decrease in HbA1c levels. A comprehensive systematic literature review and meta-analysis that included more than 170,000 obese patients treated with metabolic surgery or nonsurgical management showed that metabolic surgeries were associated with an almost 50% lower hazard of death of any cause vs. nonsurgical treatments [15]. In the subgroup of diabetic patients, the effects of metabolic surgery on survival were more pronounced than in patients without diabetes. Moreover, a recently published network meta-analysis compared different metabolic surgeries and medical therapies based on 24 trials [16]. The analysis provided evidence that surgeries achieved significantly greater reductions in HbA1c, fasting blood glucose, and BMI than medical treatment. Furthermore, improved glycaemic control was demonstrated in all three obesity classes and also in subpopulations with a baseline HbA1c below or above 8.0%. Another recently published network meta-analysis compared metabolic surgeries and nonsurgical treatment based on 17 randomized controlled trials [17]. In the network meta-analysis, all but one surgical treatments were more frequently associated with the remission of diabetes (i.e., achieving a *HbA*1*c* < 6.0%). Some studies have even confirmed the beneficial effects of bariatric surgeries on BMI and glycaemic control beyond 5 years [18–21].

A recently published systematic review collected the available outcomes of cost-effectiveness and cost-utility analysis of bariatric surgeries in patients living with obesity and T2DM compared to nonsurgical management [22]. The applied time horizon ranged from 2 years to lifetime, and the analyses were performed in most cases from the public payers' perspectives. The cost-effectiveness was declared to be at the 20,000 or 45,000 Euros/quality-adjusted life years (QALY) thresholds. Although the analyses showed significant heterogeneity in several methodological aspects (such as outcomes, comparators, and surgery types), all of the comparisons demonstrated that bariatric (metabolic) surgeries were cost-effective, and 57% of the comparisons were dominant (i.e., saved costs and resulted in improved health outcomes). Another study confirmed that bariatric surgeries were dominant over usual treatment in a morbidly obese population [23].

The objective of the current cost-effectiveness analysis was to demonstrate that bariatric surgeries are cost-effective treatment options for patients living with obesity and type 2 diabetes in Hungary using a simulation model based on the published results of randomized controlled trial.

## 2. Materials and Methods

### 2.1. Model Population

The model takes into account a subgroup of patient population [24] that is representative to Hungarian patients eligible for bariatric surgery. Patients entered the model at age 45, had a *BMI* ≥ 30 kg/m^2^, and suffered from type 2 diabetes mellitus (T2DM). The model was run in, and the outcomes were provided to three subpopulations grouped by BMI: ≥30 to 35 kg/m^2^, ≥35 to 40 kg/m^2^, and ≥40 to 50 kg/m^2^.

### 2.2. Model Structure

The current cost-effectiveness model compared SG and RYGB to standard diabetes care.

**Figure 1:**
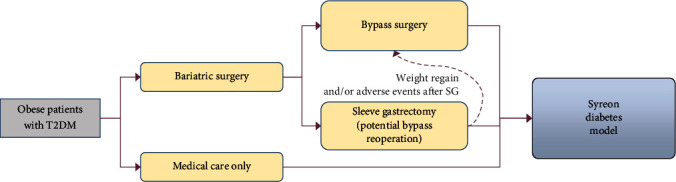
Treatment selection part of the model.

The patients entered the model at the time of their first bariatric operation (see Figure 1. All costs of preoperative medical examinations are included in the first model cycle. Moreover, all patients (whether they underwent surgery or not) moved on the patient-level simulation part of the model immediately after allocation to one of the treatment arms. The ratio of patients who underwent SG or RYGB surgeries was 60 : 40.

**Figure 2:**
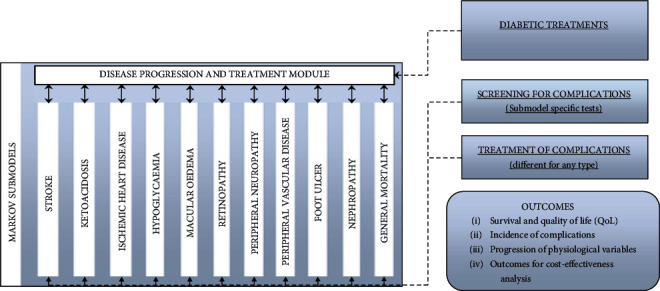
Patient-level simulation part of the model (Syreon diabetes model [24]).

The simulation part of the model was based on an earlier published diabetes model [25] which had gone through internal [26] and external validation [27]. The model consists of 10 submodels of different diabetic complications: (1) macular oedema, (2) hypoglycaemia, (3) ketoacidosis, (4) neuropathy, (5) retinopathy, (6) peripheral vascular disease, (7) nephropathy, (8) stroke, (9) foot ulcer, and (10) ischaemic heart disease. By feeding changes in HbA1c levels, BMI, systolic and diastolic blood pressure (SBP and DBP, respectively), and total (TC) and high-density lipoprotein (HDL) cholesterol levels, the model simulates the progression of these complications through a series of health states (see Figure 2). The simulation technique allows for the patients to stay simultaneously in multiple submodels, thereby allowing patients to develop multiple complications within each model cycle. In addition, the submodels are interconnected; hence, progression in one complication has the potential to influence progression in another complication. It is important to emphasize that SG patients can be converted to RYGB due to gastroesophageal reflux or weight regain.

In the economic evaluation, we modelled health outcomes for the entire lifetime of patients, and we summarized outcomes and costs in 6-month cycles. The model was prepared from the public payer's perspective and took into account the public payer's expenditures (adding out-of-pocket expenses of patients for vitamins and trace elements). The health outcomes were expressed in quality-adjusted life years (QALY) and the time spent to the first occurrence of major diabetic complications. Both costs and QALY were discounted with 3.7% that meet the requirement of the Guidelines of the Hungarian Health Economics Association [28]. The costs in Hungarian forints (HUF) were converted to Euro using the average exchange rate provided by European Central Bank (358.52 HUF/Euro) for 2021.

The ratio of differences in costs and QALYs (incremental cost-effectiveness ratio [ICER]) was used to quantify the cost-effectiveness of bariatric surgeries compared to the comparator treatment (conventional care of diabetes treatment). In cases where the bariatric surgeries resulted in cost saving (negative cost difference) and QALY gains (consequently, ICER would be represented by a negative value), the new treatment was labelled as “dominant” option (over the comparator).

### 2.3. Model Parameters

#### 2.3.1. Changes in BMI

*(1) Initial BMI Changes in the First Postoperative Year*. Based on a regression analysis of baseline BMI values and maximum BMI changes in the first postoperative year in the included studies (see Table S1 in Appendix), we found that a higher baseline BMI level corresponded to a greater BMI decrease. The initial decrease in BMI for both SG and RYGB and for each baseline BMI from 30 to 50 is summarized in Table S1 (see Appendix).

*(2) Weight Regain (BMI Rebound) up to the 6^th^ Postoperative Year*. Based on the results of the included studies (see Table S2 in Appendix), regression analyses were performed for the increase in BMI between the 2^nd^ and 6^th^ postoperative years after SG and RYGB. In one-third of the patients after SG, the weight regain was high enough (2.0 kg/m^2^/year between the 2^nd^ and 6^th^ years) to be an indication for conversion to RGYB, while in patients who did not need conversion to RYGB, a smaller weight regain was modelled (0.54 kg/m^2^/year between the 2^nd^ and 6^th^ years). The weight regain was moderate after RYGB surgery (0.84 kg/m^2^/year between the 2^nd^ and 6^th^ years; see Table S2 in Appendix). After the 6^th^ year, no further BMI increase in the surgery arm was assumed, while no BMI changes in the conventionally treated patient group were assumed for the study period.

#### 2.3.2. Changes in HbA1c Levels

*(1) Initial HbA1c Decrease in the First Postoperative Year*. Before calculating the initial changes in HbA1c levels and HbA1c rebound, baseline HbA1c levels were determined. As the baseline BMI and the baseline HbA1c levels showed a negative correlation (see Table S3 in Appendix), different baseline HbA1c levels were allocated for each of the three BMI strata. Subsequently, the initial (i.e., first year) HbA1c decrease was calculated for each BMI/HbA1c strata (see Table S4 in Appendix).

*(2) HbA1c Rebound up to the 10^th^ Postoperative Year*. When calculating HbA1c rebound between the 2^nd^ and the 10^th^ years, a continuous HbA1c increase was assumed. As the included articles showed large heterogeneity in the changes of HbA1c levels after the first postoperative 6 months, the HbA1c trajectory was modelled based on the long-term outcomes of the UKPDS study [29]. As it can be reasonably assumed that patients with reduced gastric volume and/or limited intestinal absorptive capacity would have a slower glycaemic deterioration compared to medically treated T2DM patients, half of the rate of the HbA1c increase observed in the UKPDS was assumed between the postoperative nadir and the 10^th^ year (see Table S5 in Appendix). In the conventional treatment arm, the same increase in HbA1c levels was assumed to be observed in the UKPDS study.

*(3) Conversion of SG to RYGB*. As patients after SG may suffer from GERD and/or may experience weight regain, some are reoperated via the bypass technique. The cumulative incidence of RYGB conversion is 50% in the first 15 years after SG [30, 31].

*(4) Mortality*. Hungary-specific general mortality data were taken from the Hungarian Central Statistics Office database [6]. T2DM mortality was determined via the submodels. We assumed that the events in the submodels cover most of the additional mortality risk of an obese population; therefore, no additional background mortality was added.

### 2.4. Health Outcomes

The health utility and disutility values used in the diabetes model have been previously presented [25]. Health utility and disutility values specific to obesity, changes in BMI, and the most common short- and long-term complications of bariatric surgery were derived from the literature and are summarized in Table S6 (in Appendix). The consequences (i.e., improved utilities or disutilities) of indirect effects of bariatric surgery—such as improved sexual functioning [32] or changes in diet or intake of oral medicines due to digestive disorders or drug intolerance—were not considered in the economic model.

### 2.5. Costs

The fees for surgery were calculated based on the analysis of costs of health care providers determined with the microcosting method [33], and the instrument costs were determined based on the list prices provided by the medical device manufacturer. The costs of short-term surgery adverse events were based on already established diagnosis-related groups (DRGs), whereas the costs of long-term complications were based on DRGs or outpatient care costs (including drug reimbursement expenditures; see Table S7 in the Appendix). The prevalence of surgery-related adverse events is summarized in Table S8 (in Appendix). To calculate the costs of antidiabetic treatment, we assumed that all patients used metformin. In the conventional treatment arm, we assumed that 60% of patients used insulin and 60% of patients used GLP-1RAs. The modelling of postoperative insulin treatment was based on the study by Ikramuddin et al. [34], which found that 13-18% of the operated patients remained on insulin treatment during the 5 years following surgery. In our model, the proportion of insulin-treated patients changed from 16% to 20% between 1 and 5 years postoperatively.

### 2.6. Scenario Analyses

To test the robustness of the modelling results, some assumptions were applied that theoretically reduced the advantages of bariatric surgeries over conservative diabetes treatment. The following scenarios were tested:

- Doubling of the disutilities of surgery adverse events
- Doubling of the prevalence of surgery-related adverse events
- Doubling of the rate of HbA1c rebound up to 10 years in the surgery arm
- All scenarios together with the DRG code of “gastric surgery in patients older than 18 years” (that has twice as much financing as the DRG code used in the base-case analysis)

### 2.7. Modelling Environment

The model was run in R v4.4.1, in R-Studio (2021.09.0 Build 351) environment.

## 3. Results

### 3.1. Base-Case Analysis Results

#### 3.1.1. Cost-Effectiveness

Bariatric surgeries were cost saving and resulted in improvements in health outcomes (QALY) in all three BMI classes; thus, bariatric surgeries were proven to be dominant treatment options over conservative diabetes treatment.  Table 1 summarizes the base-case analysis results with and without discounting the costs and health outcomes in the future.

#### 3.1.2. Life Expectancy and Disease-Free Survival Times

Based on modelling, bariatric surgeries extended life expectancy and delayed the investigated diabetic complications (Table 2).

### 3.2. Sensitivity Analysis Results

**Figure 3:**
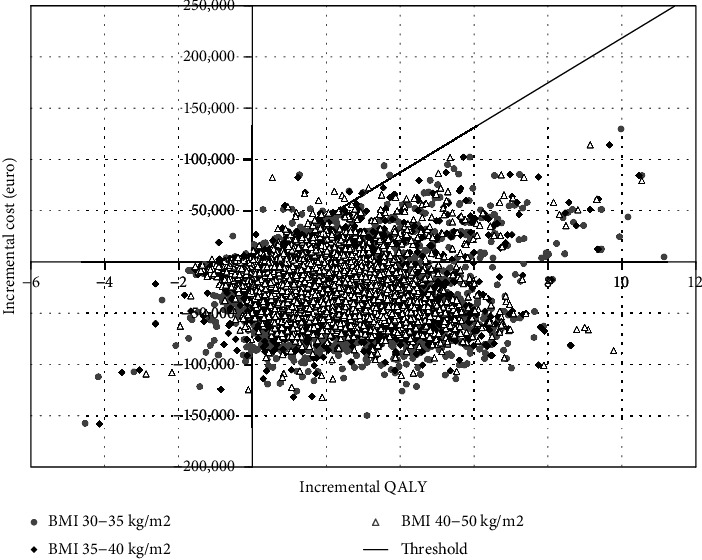
Scatter plot of the probabilistic sensitivity analysis.

The cost-effectiveness acceptability curve (Figure 3) shows that applying a 23 753 EUR/QALY willingness-to-pay threshold [28], bariatric surgery had 96%, 97%, and 98% probabilities to be cost-effective compared to conventional care of diabetes in the ≥30 to 35 kg/m^2^, the ≥35 to 40 kg/m^2^, and the ≥40 to 50 kg/m^2^ BMI ranges, respectively, and had 85%, 85%, and 87% probabilities to be dominant in the same BMI ranges.

### 3.3. Scenario Analysis Results

The results of each scenario are summarized in Table S9, Table S10, Table S11, and Table S12 in the Appendix. Bariatric surgeries remained the dominant treatment in all three BMI ranges and in all analysis scenarios, including the one scenario that applied doubled disutilities and doubled prevalence of surgery-related adverse events, doubled the rate of HbA1c rebound, and used a DRG code for bariatric surgery that had twice as much financing as the DRG code used in the base-case analysis.

## 4. Discussion

The objective of the current cost-effectiveness analysis was to demonstrate that bariatric surgeries are cost-effective treatment options for patients living with obesity and type 2 diabetes from the public payer's perspective.

The current analysis included *patients* ≥ 45 years old and with a BMI ≥30 kg/m^2^ and compared the bariatric surgeries that were most frequently used in Hungary to conservative diabetes treatment. The health outcomes were expressed as quality-adjusted life years, and the cost calculations were based on the expenses that the public payer paid for specific inpatient and outpatient care.

The analysis confirmed that the selected bariatric surgeries (i.e., sleeve gastrectomy (SG) and Roux-en-Y gastric bypass (RYGB)) can achieve improved health outcomes while saving costs in obese patients with T2DM (i.e., “dominant” treatment options) compared to conventional diabetes treatment. This “dominance” was confirmed in all three BMI categories. In addition, these bariatric surgeries extended the time to the first appearance of most diabetic complications. The robustness of the analysis was supported by the results of the scenario analyses that confirmed the results of the base-case analysis: SG and RYGB proved to be cost saving and QALY improving treatments even when assumptions were made that worsened the effectiveness or increased the costs of the surgeries.

It is important to emphasize that this cost-effectiveness analysis used a hypothetical DRG weight that was calculated in 2017 based on hospital microcosting. Although the DRG weight would likely be higher in 2022, it is unlikely that this increased surgery cost (public payer funding) may jeopardize the cost-effectiveness of bariatric surgeries. In addition, our analysis did not investigate the metabolic consequences of obesity other than diabetes (e.g., the lipid profile) or the nonmetabolic consequences (e.g., damages to the musculoskeletal system, increased risk of complications in general surgical anaesthesia, worse self-esteem, and worse quality of life in social and psychological domains); however, it can reasonably be assumed that the inclusion of these pathologies in the cost-effectiveness analysis would further increase the dominance of bariatric surgeries.

Our results are consistent with those of other analyses. In the systematic review by Jordan et al., all of the collected cost-effectiveness analyses confirmed that bariatric surgeries were cost-effective compared to conventional diabetes treatment, and more than half of the analyses were dominant [22]. Similar to the outcomes of the meta-analysis by Syn et al., our model showed longer life expectancy in those patients who underwent bariatric surgery [15], although the increase in life expectancy was less than that in the analysis by Syn et al. The possible reason for the difference is that our model only considered diabetes-related death, whereas the studies included in the analysis by Syn et al. considered death for any reason.

The main limitation of our analysis was that it only considered the consequences of diabetes. Although it can reasonably be expected that the inclusion of other metabolic and nonmetabolic consequences of obesity would further increase the improvement in the achieved health outcomes and the costs saved by bariatric surgeries (compared to conservative diabetes treatment), a sole focus on diabetes slightly decreased the validity of the analysis.

In conclusion, our analysis provided strong evidence that sleeve gastrectomy and Roux-en-Y gastric bypass are more effective alternatives than conventional diabetes treatment in patients living with obesity and type 2 diabetes. In addition, these treatments can save on long-term public payer expenses. Therefore, it can be strongly recommended that a wider population of patients living with obesity and type 2 diabetes be able to have access to these surgeries in Hungary.

## Figures and Tables

**Table 1:** Base-case analysis results.

BMI classes	Values with no discounting costs and health outcomes			Values with discounting both costs and health outcomes^†^			Cost-effectiveness evaluation
	Bariatric surgery	Comparator	Differences	Bariatric surgery	Comparator	Differences	
BMI: 30-34.99 kg/m^2^							
Cost (Euro)	124 362	171 135	-46 773	67 035	91 419	-24 384	Dominant
QALY	12.94	10.13	2.81	8.51	7.01	1.50	
BMI: 35-39.99 kg/m^2^							
Cost (Euro)	120 839	159 080	-38 241	65 341	84 112	-18 771	Dominant
QALY	12.62	10.01	2.61	8.29	6.93	1.36	
BMI: 40-50 kg/m^2^							
Cost (Euro)	107 294	138 673	-31 379	64 867	81 932	-17 064	Dominant
QALY	11.91	9.11	2.80	7.85	6.38	1.47	

^†^3.7% discounting for both costs and QALY in line with the Hungarian Health Economic Guidelines [27].

**Table 2:** Mean life expectancy and disease-free survival time of diabetes complications in the analysis arms (years).

BMI classes	Bariatric surgeries	Comparator	Differences
BMI: 30-34.99 kg/m^2^			
Life expectancy	28.84	27.53	1.31
Stroke	23.78	21.61	2.16
Myocardial infarction	26.25	23.34	2.91
Renal transplant	28.74	27.44	1.30
Blindness	22.12	19.38	2.74
Leg amputation	21.31	19.87	1.43
Any disease	14.44	11.89	2.55
BMI: 35-39.99 kg/m^2^			
Life expectancy	29.03	28.01	1.02
Stroke	24.04	22.38	1.66
Myocardial infarction	26.54	24.28	2.26
Renal transplant	28.93	27.92	1.00
Blindness	22.66	20.78	1.88
Leg amputation	21.64	20.43	1.21
Any disease	14.85	12.94	1.91
BMI: 40-50 kg/m^2^			
Life expectancy	29.10	28.19	0.91
Stroke	24.16	22.72	1.44
Myocardial infarction	26.64	24.70	1.94
Renal transplant	28.99	28.10	0.90
Blindness	22.84	21.39	1.45
Leg amputation	21.80	20.77	1.03
Any disease	15.00	13.46	1.55

## Data Availability

The datasets supporting the conclusions of this article are included in the main text and in its supplementary materials, including model description and input data with data sources. All other datasets used and analysed in the current study are available from the corresponding author upon request.

## Supplementary Materials

Supplementary Materials Table S1 to Table S12 and their related references with separate consecutive numbering are provided in a separate file.
