# Supplementary material for: Cost-Effectiveness of Bariatric Surgery in Patients Living with Obesity and Type 2 Diabetes
Source: J Diabetes Res. 2023 Dec 16;2023:9686729. doi: 10.1155/2023/9686729 (PMC10748723; doi:10.1155/2023/9686729)
Supplement: Supplementary Materials — Table S1 to Table S12 and their related references with separate consecutive numbering are provided in a separate file. [file 9686729.f1.docx]

**Cost-effectiveness of bariatric surgery in patients living with obesity and type 2 diabetes**

# Appendix

Table S1. Initial changes in BMI in the first postoperative year depending on the baseline BMI and the type of surgery performed (regression analysis results)

| **Pre-operative BMI** | **BMI decrease (kg/m^2^)** | | **Sources** |
| --- | --- | --- | --- |
|  | **SG** | **RYGB** |  |
| 30 | -5.0 | -5.7 | ^1-9^ |
| 31 | -5.3 | -6.2 |  |
| 32 | -5.7 | -6.7 |  |
| 33 | -6.1 | -7.2 |  |
| 34 | -6.4 | -7.7 |  |
| 35 | -6.8 | -8.2 |  |
| 36 | -7.1 | -8.7 |  |
| 37 | -7.5 | -9.2 |  |
| 38 | -7.8 | -9.7 |  |
| 39 | -8.2 | -10.2 |  |
| 40 | -8.5 | -10.6 |  |
| 41 | -8.9 | -11.1 |  |
| 42 | -9.2 | -11.6 |  |
| 43 | -9.6 | -12.1 |  |
| 44 | -9.9 | -12.6 |  |
| 45 | -10.3 | -13.1 |  |
| 46 | -10.7 | -13.6 |  |
| 47 | -11.0 | -14.1 |  |
| 48 | -11.4 | -14.6 |  |
| 49 | -11.7 | -15.1 |  |
| 50 | -12.1 | -15.6 |  |

Table S2. Weight regain (BMI rebound) between the 2^nd^ and the 6^th^ postoperative years after the maximal weight loss (regression analysis results)

| **Postoperative years** | **BMI increase after nadir (kg/m^2^)** | | | **Sources** |
| --- | --- | --- | --- | --- |
|  | **SG without weight regain** | **SG with weight regain** | **RYGB** |  |
| 2 | 0.5 | 2.0 | 0.8 | ^2,3,5-14^, expert opinion |
| 3 | 1.1 | 4.0 | 1.7 |  |
| 4 | 1.6 | 6.0 | 2.5 |  |
| 5 | 2.2 | 8.0 | 3.4 |  |
| 6 | 2.7 | 10.0 | 4.2 |  |

Table S3. Baseline (preoperative) HbA1c levels by baseline BMI ranges

| **Baseline BMI ranges (kg/m^2^)** | **Baseline HbA1c levels (%)** | **Sources** |
| --- | --- | --- |
| 30-34.9 | 9.5 | ^1-12,15-22^ |
| 35-39.9 | 8.4 |  |
| 40-50 | 7.7 |  |

Table S4. Initial decrease of HbA1c levels in the first postoperative year by baseline HbA1c levels

| **Baseline HbA1c levels (%)** | **Initial decrease in HbA1c levels (%)** | | **Sources** |
| --- | --- | --- | --- |
|  | **SG** | **RYGB** |  |
| 9.5 | -2.4 | -3.1 | ^2,5,7,9,11^ |
| 8.4 | -1.8 | -2.2 |  |
| 7.7 | -1.5 | -1.7 |  |

Table S5. BMI rebound between the 2^nd^ and the 10^th^ postoperative years after the maximal BMI decrease

| **Time horizon** | **Initial decrease in HbA1c levels (%/year)** | | **Source** |
| --- | --- | --- | --- |
|  | **In the surgery patients** | **In the conventionally treated patients** |  |
| Between the 2^nd^ and the 10^th^ years after the surgery | 0.1 | 0.2 | ^23^ |

Table S6. Utility values used in the bariatric surgery specific health states

| **Health states** | **Disutility** | **Sources** | **Comments** |
| --- | --- | --- | --- |
| Mean change of utility with 1 unit increase in BMI | -0.0056 | ^24^ |  |
| Minor bleeding | -0.0016 | ^25^ |  |
| Major bleeding | -0.1933 | ^25,26^ | Major bleeding disutility plus intensive care disutility in 50% of the cases |
| Intestinal leak | -0.25 | ^26,27^ | Supposing 50% laparoscopic or open surgery plus intensive care treatment in 50% of the cases |
| Intestinal perforation | -0.25 | ^26,27^ | Supposing 50% laparoscopic or open surgery plus intensive care treatment in 50% of the cases |
| Intestinal obstruction | -0.14 | ^26,27^ | Supposing 50% laparoscopic or open surgery plus intensive care treatment in 20% of the cases |
| Cholelithiasis | -0.079 | ^27,28^ | Cholelithiasis disutility plus laparoscopic surgery disutility |
| Internal hernia | -0.128 | ^26,27^ | Supposing 80% laparoscopic or 20% open surgery plus intensive care treatment in 20% of the cases |
| Abdominal wall hernia | -0.083 | ^27,28^ | Hernia disutility plus laparoscopic surgery disutility |
| Abdominal abscess | -0.25 | ^26,27^ | Supposing 50% laparoscopic or open surgery plus intensive care treatment in 50% of the cases |
| GERD (mixed population of patients with moderate and severe GERD) | -0.15 | ^29^ | Assuming 80% moderate and 20% severe GERD |
| Barrett’s oesophagus | -0.15 | ^29^ | Assuming 80% moderate and 20% severe GERD |
| Oesophageal cancer | -0.2364 | ^30^ | Assuming a mixed population of patients with early (45%) or late stage (45%) or with distant metastasis (10%) oesophageal cc. |

Table S7. Cost of bariatric surgery specific health states

| Cost items | Cost (Euro) | Source | Comments |
| --- | --- | --- | --- |
| **Bariatric surgeries** | | | |
| Preoperative investigations | 125 | ^31^ | Including laboratory blood tests, abdominal ultrasound examination, esophago-gastro-duodenoscopy, consultation with cardiologist and psychologist, lung function tests |
| Surgery instruments | 3 025 | list prices of a medical device manufacturer | Same instrument costs for both sleeve and bypass surgeries |
| Surgery (DRG-based financing) | 1 030 | ^32^ |  |
| *Total* | *4 055* |  |  |
| **Postoperative routine care** | | | |
| In the first postoperative year | 250/year | ^31^ | Including laboratory blood tests (4 times per year) surgical examinations (4 times per year), abdominal ultrasound (once per year) |
| Yearly from the second postoperative year | 65/year |  | Including laboratory blood tests (once per year) surgical examinations (once per year), abdominal ultrasound (once per year) |
| Multivitamin with trace elements and iron | 223/year |  |  |
| **Adverse events** | | | |
| Minor bleeding | 647/event | ^31^ | Inpatient care costs based on DRG-based reimbursement system |
| Major bleeding | 3 169/event |  |  |
| Intestinal leak | 3 169/event |  |  |
| Intestinal perforation | 2 028/event |  |  |
| Intestinal obstruction | 1 511/event |  |  |
| Cholelithiasis | 824/event |  |  |
| Internal hernia | 1 511/event |  |  |
| Incisional (abdominal) hernia | 1 231/event |  |  |
| Abdominal abscess | 2 028/event |  |  |
| Oesophageal cc | 4 676/event |  |  |
| GERD | 40/person-year |  | Costs of daily use of proton-pump inhibitors |
| Barrett’s oesophagus | 52/person-year |  | Costs of yearly esophago-gastro-duodenoscopy and of daily use of proton-pump inhibitors |

Table S8. Prevalence of surgery adverse events by surgery types

|  |  | **Prevalence in the postoperative periods** | | | **Sources** |
| --- | --- | --- | --- | --- | --- |
| **Health states** | Surgery type | <6 months postoperatively | Between 6 & 12 months postoperatively | >1 year postoperatively |  |
| Bleeding, minor | SG | 4% | 3% | 3% | ^8,9,33-35^, expert opinion |
|  | RYGB | 4% | 0% | 0% |  |
| Bleeding, major | SG | 1% | 0.5% | 0.5% |  |
|  | RYGB | 1% | 0.5% | 0.5% |  |
| Perforation | SG | 0% | 0.5% | 0.5% |  |
|  | RYGB | 0% | 0.5% | 0.5% |  |
| Intestinal leak | SG | 4% | 0% | 0% |  |
|  | RYGB | 4% | 0% | 0% |  |
| Intestinal obstruction | SG | 0% | 0% | 0% |  |
|  | RYGB | 4% | 5% | 2% |  |
| Cholelithiasis | SG | 0% | 5% | 15-20% |  |
|  | RYGB | 0% | 5% | 15-20% |  |
| Internal hernia | SG | 0% | 0% | 0% |  |
|  | RYGB | 0% | 1-2% | 0% |  |
| Incisional hernia | SG | 0% | 5% | 5% |  |
|  | RYGB | 0% | 5% | 5% |  |
| Intra-abdominal abscess | SG | 1% | 0% | 0% |  |
|  | RYGB | 1% | 0% | 0% |  |
| GERD | SG | 0% | 30% | 50% |  |
| Barrett’s oesophagus | SG | 0% | 0% | 20% |  |
| Oesophageal cc | SG | 0% | 0% | 0.08%/patient-year |  |

Table S9. Analysis results with doubled disutilities of surgery adverse events

| **BMI classes** | **Bariatric surgery** | **Comparator** | **Differences** | **Cost-effectiveness evaluation** |
| --- | --- | --- | --- | --- |
| **BMI: 30-34.99 kg/m^2^** | | | | |
| Cost (Euro) | 68 138 | 91 416 | -23 277 | **Dominant** |
| QALY | 8.22 | 7.01 | 1.21 |  |
| **BMI: 35-39.99 kg/m^2^** | | | | |
| Cost (Euro) | 66 440 | 84 114 | -17 674 | **Dominant** |
| QALY | 7.98 | 6.93 | 1.05 |  |
| **BMI: 40-50 kg/m^2^** | | | | |
| Cost (Euro) | 59 124 | 73 424 | -14 300 | **Dominant** |
| QALY | 7.53 | 6.38 | 1.15 |  |

Table S10. Analysis results with doubled prevalence of surgery adverse events

| **BMI classes** | **Bariatric surgery** | **Comparator** | **Differences** | **Cost-effectiveness evaluation** |
| --- | --- | --- | --- | --- |
| **BMI: 30-34.99 kg/m^2^** | | | | |
| Cost (Euro) | 70 806 | 91 438 | -20 631 | **Dominant** |
| QALY | 8.71 | 7.01 | 1.70 |  |
| **BMI: 35-39.99 kg/m^2^** | | | | |
| Cost (Euro) | 69 017 | 84 077 | -15 060 | **Dominant** |
| QALY | 8.51 | 6.93 | 1.58 |  |
| **BMI: 40-50 kg/m^2^** | | | | |
| Cost (Euro) | 68 464 | 81 906 | -13 442 | **Dominant** |
| QALY | 8.10 | 6.38 | 1.73 |  |

Table S11. Analysis results with double rate of HbA1c rebound

| **BMI classes** | **Bariatric surgery** | **Comparator** | **Differences** | **Cost-effectiveness evaluation** |
| --- | --- | --- | --- | --- |
| **BMI: 30-34.99 kg/m^2^** | | | | |
| Cost (Euro) | 72 535 | 91 370 | -18 835 | **Dominant** |
| QALY | 8.32 | 7.01 | 1.31 |  |
| **BMI: 35-39.99 kg/m^2^** | | | | |
| Cost (Euro) | 70 560 | 84 127 | -13 566 | **Dominant** |
| QALY | 8.10 | 6.92 | 1.18 |  |
| **BMI: 40-50 kg/m^2^** | | | | |
| Cost (Euro) | 69 853 | 81 960 | -12 107 | **Dominant** |
| QALY | 7.67 | 6.37 | 1.29 |  |

Table S12. Analysis results with doubled disutilities and prevalence of surgery adverse events, doubled HbA1c rebound rate together with the DRG code of “Gastric surgery in patients older than 18 years” (that has twice as much financing as the DRG code used in the base-case analysis)

| **BMI classes** | **Bariatric surgery** | **Comparator** | **Differences** | **Cost-effectiveness evaluation** |
| --- | --- | --- | --- | --- |
| **BMI: 30-34.99 kg/m^2^** | | | | |
| Cost (Euro) | 75 575 | 91 392 | -15 817 | **Dominant** |
| QALY | 8.19 | 7.01 | 1.18 |  |
| **BMI: 35-39.99 kg/m^2^** | | | | |
| Cost (Euro) | 73 491 | 84 117 | -10 627 | **Dominant** |
| QALY | 7.99 | 6.92 | 1.06 |  |
| **BMI: 40-50 kg/m^2^** | | | | |
| Cost (Euro) | 72 769 | 81 970 | -9 200 | **Dominant** |
| QALY | 7.58 | 6.38 | 1.21 |  |

# References in Appendix

1. Abbatini F, Capoccia D, Casella G, Soricelli E, Leonetti F, Basso N. Long-term remission of type 2 diabetes in morbidly obese patients after sleeve gastrectomy. Surg Obes Relat Dis. Jul-Aug 2013;9(4):498-502.

2. Brethauer SA, Aminian A, Romero-Talamas H, et al. Can diabetes be surgically cured? Long-term metabolic effects of bariatric surgery in obese patients with type 2 diabetes mellitus. Ann Surg. Oct 2013;258(4):628-36; discussion 636-7.

3. Keidar A, Hershkop KJ, Marko L, et al. Roux-en-Y gastric bypass vs sleeve gastrectomy for obese patients with type 2 diabetes: a randomised trial. Diabetologia. Sep 2013;56(9):1914-8.

4. Lee WJ, Chong K, Lin YH, Wei JH, Chen SC. Laparoscopic sleeve gastrectomy versus single anastomosis (mini-) gastric bypass for the treatment of type 2 diabetes mellitus: 5-year results of a randomized trial and study of incretin effect. Obes Surg. Sep 2014;24(9):1552-62.

5. Mas-Lorenzo A, Benaiges D, Flores-Le-Roux JA, et al. Impact of different criteria on type 2 diabetes remission rate after bariatric surgery. Obes Surg. Nov 2014;24(11):1881-7.

6. Dicker D, Yahalom R, Comaneshter DS, Vinker S. Long-Term Outcomes of Three Types of Bariatric Surgery on Obesity and Type 2 Diabetes Control and Remission. Obes Surg. Aug 2016;26(8):1814-20.

7. Casajoana A, Pujol J, Garcia A, et al. Predictive Value of Gut Peptides in T2D Remission: Randomized Controlled Trial Comparing Metabolic Gastric Bypass, Sleeve Gastrectomy and Greater Curvature Plication. Obes Surg. Sep 2017;27(9):2235-2245.

8. Schauer PR, Bhatt DL, Kirwan JP, et al. Bariatric Surgery versus Intensive Medical Therapy for Diabetes - 5-Year Outcomes. N Engl J Med. Feb 16 2017;376(7):641-651.

9. Murphy R, Clarke MG, Evennett NJ, et al. Laparoscopic Sleeve Gastrectomy Versus Banded Roux-en-Y Gastric Bypass for Diabetes and Obesity: A Prospective Randomised Double-Blind Trial. Obes Surg. Feb 2018;28(2):293-302.

10. Pournaras DJ, Aasheim ET, Sovik TT, et al. Effect of the definition of type II diabetes remission in the evaluation of bariatric surgery for metabolic disorders. Br J Surg. Jan 2012;99(1):100-3.

11. Yang J, Wang C, Cao G, et al. Long-term effects of laparoscopic sleeve gastrectomy versus Roux-en-Y gastric bypass for the treatment of Chinese type 2 diabetes mellitus patients with body mass index 28-35 kg/m2. BMC Surg. Jul 2015;15:88.

12. Tang Q, Sun Z, Zhang N, et al. Cost-Effectiveness of Bariatric Surgery for Type 2 Diabetes Mellitus: A Randomized Controlled Trial in China. Medicine (Baltimore). May 2016;95(20): e3522.

13. Felsenreich DM, Ladinig LM, Beckerhinn P, et al. Update: 10 Years of Sleeve Gastrectomy-the First 103 Patients. Obes Surg. Nov 2018;28(11):3586-3594.

14. Felsenreich DM, Artemiou E, Steinlechner K, et al. Fifteen Years After Sleeve Gastrectomy: Weight Loss, Remission of Associated Medical Problems, Quality of Life, and Conversions to Roux-en-Y Gastric Bypass-Long-Term Follow-Up in a Multicenter Study. Obes Surg. Aug 2021;31(8):3453-3461.

15. Kim S, Richards WO. Long-term follow-up of the metabolic profiles in obese patients with type 2 diabetes mellitus after Roux-en-Y gastric bypass. Ann Surg. Jun 2010;251(6):1049-55.

16. Liang Z, Wu Q, Chen B, Yu P, Zhao H, Ouyang X. Effect of laparoscopic Roux-en-Y gastric bypass surgery on type 2 diabetes mellitus with hypertension: a randomized controlled trial. Diabetes Res Clin Pract. Jul 2013;101(1):50-6.

17. Courcoulas AP, Belle SH, Neiberg RH, et al. Three-Year Outcomes of Bariatric Surgery vs Lifestyle Intervention for Type 2 Diabetes Mellitus Treatment: A Randomized Clinical Trial. JAMA Surg. Oct 2015;150(10):931-40.

18. Mingrone G, Panunzi S, De Gaetano A, et al. Bariatric-metabolic surgery versus conventional medical treatment in obese patients with type 2 diabetes: 5 year follow-up of an open-label, single-centre, randomised controlled trial. Lancet. Sep 2015;386(9997):964-73.

19. Cummings DE, Arterburn DE, Westbrook EO, et al. Gastric bypass surgery vs intensive lifestyle and medical intervention for type 2 diabetes: the CROSSROADS randomised controlled trial. Diabetologia. May 2016;59(5):945-53.

20. Ikramuddin S, Korner J, Lee WJ, et al. Lifestyle Intervention and Medical Management With vs Without Roux-en-Y Gastric Bypass and Control of Hemoglobin A1c, LDL Cholesterol, and Systolic Blood Pressure at 5 Years in the Diabetes Surgery Study. JAMA. Jan 2018;319(3):266-278.

21. Simonson DC, Halperin F, Foster K, Vernon A, Goldfine AB. Clinical and Patient-Centered Outcomes in Obese Patients with Type 2 Diabetes 3 Years After Randomization to Roux-en-Y Gastric Bypass Surgery Versus Intensive Lifestyle Management: The SLIMM-T2D Study. Diabetes Care. Apr 2018;41(4):670-679.

22. Mingrone G, Panunzi S, De Gaetano A, et al. Metabolic surgery versus conventional medical therapy in patients with type 2 diabetes: 10-year follow-up of an open-label, single-centre, randomised controlled trial. Lancet. Jan 23 2021;397(10271):293-304.

23. UKPDS Group. Intensive blood-glucose control with sulphonylureas or insulin compared with conventional treatment and risk of complications in patients with type 2 diabetes (UKPDS 33). UK Prospective Diabetes Study (UKPDS) Group. Lancet. Sep 1998;352(9131):837-53.

24. Hoerger TJ, Zhang P, Segel JE, Kahn HS, Barker LE, Couper S. Cost-effectiveness of bariatric surgery for severely obese adults with diabetes. Diabetes Care. Sep 2010;33(9):1933-9.

25. Doble B, Pufulete M, Harris JM, et al. Health-related quality of life impact of minor and major bleeding events during dual antiplatelet therapy: a systematic literature review and patient preference elicitation study. Health Qual Life Outcomes. Sep 2018;16(1):191.

26. Jiang M, Leung NH, Ip M, You JHS. Cost-effectiveness analysis of ribotype-guided fecal microbiota transplantation in Chinese patients with severe Clostridium difficile infection. PLoS One. 2018;13(7): e0201539.

27. Krog AH, Sahba M, Pettersen EM, Wisloff T, Sundhagen JO, Kazmi SS. Cost-utility analysis comparing laparoscopic vs open aortobifemoral bypass surgery. Vasc Health Risk Manag. 2017;13:217-224.

28. Sullivan PW, Ghushchyan V. Preference-Based EQ-5D index scores for chronic conditions in the United States. Med Decis Making. Jul-Aug 2006;26(4):410-20.

29. Kartman B, Gatz G, Johannesson M. Health state utilities in gastroesophageal reflux disease patients with heartburn: a study in Germany and Sweden. Med Decis Making. Jan-Feb 2004;24(1):40-52.

30. Gordon LG, Mayne GC, Hirst NG, et al. Cost-effectiveness of endoscopic surveillance of non-dysplastic Barrett's esophagus. Gastrointest Endosc. Feb 2014;79(2):242-56 e6.

31. NHIF. National Institute of Health Insurance Fund, Financing Databases. <http://www.neak.gov.hu/felso_menu/szakmai_oldalak/gyogyito_megeleozo_ellatas/adatbazisok/torzsek/torzsek>. Accessed September 30, 2022

32. Dózsa C, Illés L, Paszt A, Mohos E. A bariátriai sebészet társadalombiztosítási finanszírozásának szükségessége Magyarországon [The need for public payer's financing of bariatric surgery in Hungary]. IME - Journal of Hungarian Interdisciplinary Medicine. 2018;17(10):18.

33. Boza C, Gamboa C, Salinas J, Achurra P, Vega A, Perez G. Laparoscopic Roux-en-Y gastric bypass versus laparoscopic sleeve gastrectomy: a case-control study and 3 years of follow-up. Surg Obes Relat Dis. May-Jun 2012;8(3):243-9.

34. Zhang Y, Zhao H, Cao Z, et al. A randomized clinical trial of laparoscopic Roux-en-Y gastric bypass and sleeve gastrectomy for the treatment of morbid obesity in China: a 5-year outcome. Obes Surg. Oct 2014;24(10):1617-24.

35. Salminen P, Helmio M, Ovaska J, et al. Effect of Laparoscopic Sleeve Gastrectomy vs Laparoscopic Roux-en-Y Gastric Bypass on Weight Loss at 5 Years Among Patients with Morbid Obesity: The SLEEVEPASS Randomized Clinical Trial. JAMA. Jan 16 2018;319(3):241-254.
